# Metabolic acidosis post kidney transplantation

**DOI:** 10.3389/fphys.2022.989816

**Published:** 2022-08-23

**Authors:** Hafsa Tariq, Mirela Dobre

**Affiliations:** ^1^ Division of Nephrology, University of Rochester Medical Center, Rochester, NY, United States; ^2^ Division of Nephrology and Hypertension, University Hospitals Cleveland Medical Center, Case Western Reserve University, Cleveland, OH, United States

**Keywords:** metabolic acidosis, kidney transplant, renal tubular acidosis, tubulointerstial injury, graft failure

## Abstract

Metabolic acidosis, a common complication in patients with chronic kidney disease (CKD), results in a multitude of deleterious effects. Though the restoration of kidney function following transplantation is generally accompanied by a correction of metabolic acidosis, a subset of transplant recipients remains afflicted by this ailment and its subsequent morbidities. The vulnerability of kidney allografts to metabolic acidosis can be attributed to reasons similar to pathogenesis of acidosis in non-transplant CKD, and to transplant specific causes, including donor related, recipient related, immune mediated factors, and immunosuppressive medications. Correction of metabolic acidosis in kidney transplantation either with alkali therapy or through dietary manipulations may have potential benefits and the results of such clinical trials are eagerly awaited. This review summarizes the published evidence on the pathogenesis and clinical consequences of chronic metabolic acidosis in kidney transplant recipients.

## Introduction

Metabolic acidosis, defined as serum total CO_2_ level below 22mEq/L is a common complication in advanced chronic kidney diseases (CKD), and there is a graded association with CKD severity (Dobre et al., 2013). Kidney transplantation, the preferred treatment modality for kidney replacement therapy is associated with restoration of several essential kidney functions, however acid base abnormalities are slow to correct. It is estimated that the prevalence of metabolic acidosis ranges from 12% to 58% (Park et al., 2017; Djamali et al., 2019) in kidney transplant recipients and it can occur at a relatively higher estimated glomerular filtration rate (eGFR) when compared to general population with non-transplant CKD. This is primarily related to the inability of the single functioning kidney to excrete the daily acid load, added to the tubular toxicity of the immunosuppressive medications, particularly calcineurin inhibitors. Recipient and donor characteristics have been postulated to also play a role in the development of mild to moderate degrees of chronic metabolic acidosis after kidney transplantation.

Persistent metabolic acidosis can cause several complications. These include, but are not limited to the development of metabolic bone disease, sarcopenia, insulin resistance, anemia, kidney disease progression, and increased all-cause mortality in individuals with non-transplant CKD (Dobre et al., 2013). It is reasonable to assume that the same consequences will be encountered in metabolic acidosis occurring after kidney transplantation, though the evidence is somewhat limited. The association with eGFR decline and allograft loss has been described (Park et al., 2017), as well as the association with cardiovascular morbidity and mortality, although the evidence remains scarce (Djamali et al., 2019; Bohling et al., 2021).

In this review, we describe the proposed mechanisms of the development of metabolic acidosis in kidney transplant recipients, and detail the factors pertinent to chronic kidney disease in general, and those specific to kidney transplantation. We also discuss the complications associated with chronic metabolic acidosis and the available evidence in support of its treatment in kidney transplant recipients.

## Epidemiology

The actual prevalence of metabolic acidosis in kidney transplant recipients remains unclear, primarily due to multiple definitions being used and the timing relative to kidney transplantation event. Evidence for impaired tubular hydrogen ion transport, and diminished renal ammonia production was mechanistically studied and reported for the first time in 1967 in a transplant patient with multiple tubular defects (Massry et al., 1967). The presence of renal tubular acidosis alone has been described in multiple studies since then, most of the data obtained in the early post kidney transplantation period (Massry et al., 1967; Better et al., 1970; Wilson and Siddiqui, 1973). More recently, in cross-sectional studies, the incidence of renal tubular acidosis has been reported up to 33–35% of kidney transplant recipients (Keven et al., 2007; Malik et al., 2011).

In a cross sectional study of 823 individuals with a kidney transplant, 58% of participants had a sodium bicarbonate concentration less than 24 mmol/L (Yakupoglu et al., 2007) In a large retrospective cohort of 2318 kidney transplant recipients (Park et al., 2017), almost 14% of participants with eGFR 30–60 ml/min/1.73 m (Park et al., 2017), had metabolic acidosis at six months post-transplant, and this remained relatively persistent at 60 months post-transplant with 16% of participants having metabolic acidosis. Amongst the individuals with eGFR 15–30 ml/min/1.73 m (Park et al., 2017), the prevalence of metabolic acidosis was much higher, estimated at 63% at 6 months, and 60% at 60 months post-transplant. In a post hoc analysis of an open label randomized study of 90 kidney transplant recipients, the prevalence of metabolic acidosis was as high as 63% immediately after the transplantation. This decreased to 28% at 12 months post-transplant as the eGFR increased (Wiegand et al., 2019).

## Pathophysiology

**FIGURE 1:**
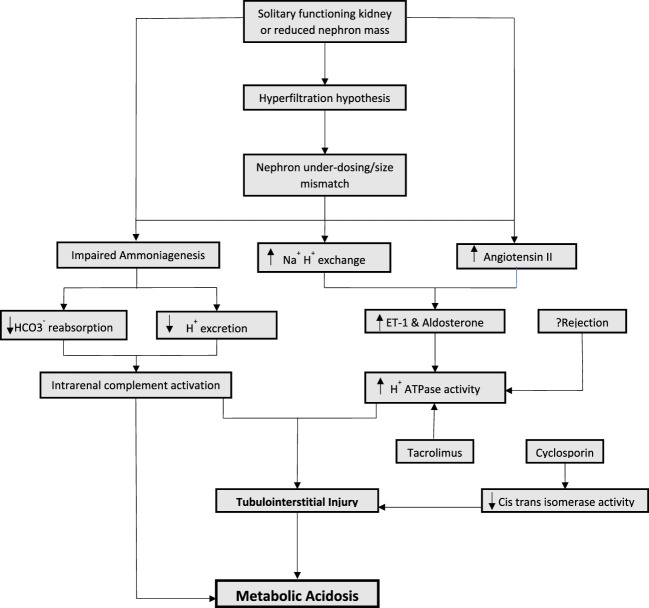
Mechanisms of metabolic acidosis in kidney transplantation.

A healthy kidney maintains acid-base homeostasis primarily by ammoniagenesis, regeneration of bicarbonate and excretion of hydrogen ions (Kraut and Madias, 2016). As the kidney function is lost and replaced by the solitary kidney allograft, the amount of ammonia production per nephron increases as a compensatory mechanism. This gives rise to hyperchloremic (non-anion gap) metabolic acidosis. However, the excretion of urinary acid continues to decrease. The increase ammonia production leads to a vicious cycle involving intrarenal complement activation, which results in tubulointerstitial injury. This initiates a cascade of events reducing the kidney’s capacity to synthesize ammonia and further decreasing urinary acid excretion (Clark et al., 1990; Raphael et al., 2017; Raphael, 2018) (Figure 1). Accumulation of phosphate, sulfate, and other non-volatile acids takes place eventually leading to high anion gap metabolic acidosis, as the allograft function is progressively lost.

Increased endogenous endothelin has been proposed to play a role in the progression of kidney disease in animal models (Wesson, 2001). Endothelin-1 (ET-1) is known to promote acid excretion by enhancing Na+/H+ exchange in proximal and distal tubules which reduces bicarbonate secretion and stimulates aldosterone. Consequently, the increase in aldosterone leads to an increase in H + ATPase activity, resulting in inflammation and tubulointerstitial injury (Wesson, 2001; Bento et al., 2007; Wesson et al., 2011; Wesson et al., 2015). In rat models who underwent subtotal nephrectomy, increased levels of systemic and renal endothelin-1 were observed (Wesson, 2001). Despite normal serum bicarbonate, intra renal acid accumulation and renal angiotensin II levels are increased which result in increased ET-1 and aldosterone levels, leading to tubular toxicity. It is important to note that angiotensin II receptor antagonism alone was not as effective for preserving eGFR decline compared to alkali supplementation, which resulted in decreasing intra renal acid accumulation (Wesson, 2001; Wesson et al., 2011; Wesson et al., 2015).

The mechanism of nephron hyperfiltration and compensatory increase in ammoniagenesis leading to complement activation and increased endothelin and aldosterone is especially important in kidney transplant recipients. Hyperfiltration hypothesis has been well described in kidney transplant recipients (Terasaki et al., 1994). By virtue of having a solitary functioning kidney and therefore, reduced nephron mass, the mechanisms of metabolic acidosis post kidney transplantation can be extrapolated from observations derived from animal models with subtotal nephrectomies. In addition to having a solitary kidney, factors pertinent to donor and recipient characteristics also play a significant role in nephron hyperfiltration (Keven et al., 2007; Kocyigit et al., 2010).

The concept of “nephron underdosing” was described by Brenner in 1993 as a potential cause for long term allograft failure (Brenner and Milford, 1993). It emphasized the importance of size mismatch between the donor and recipient. Hyperfiltration hypothesis was later described by Terasaki, demonstrating that reduced renal mass due to donor-recipient size mismatch results in compensatory nephron hypertrophy and nephron burn out resulting in chronic allograft failure (Terasaki et al., 1994). This is probably true for both living and deceased donor recipients. Reduced nephron mass can lead to proportionately reduced excretion of urinary acid, therefore contributing to development of acidosis.

Immunosuppressive medications, most notably, calcineurin inhibitors play an important role in the development of metabolic acidosis in transplant recipients. Unlike acidosis seen with nephron hypertrophy, that can be either non-anion gap or high anion gap acidosis, calcineurin inhibitors are typically associated with renal tubular acidosis (RTA) (Watanabe et al., 2005; Mohebbi et al., 2009). Calcineurin inhibitors (CNIs) are known to have a dose and duration dependent effect causing renal tubular toxicity. This tubular toxicity resulting in RTA is hypothesized to be more functional rather than structural. Hyperchloremic (non-anion gap) metabolic acidosis associated with CNI administration develops only in the setting of increased intrarenal post-glomerular blood flow and has been observed to be accompanied by the CNI arteriolopathy (Kaneko et al., 2022). Several mechanism of CNI induced hyperchloremic metabolic acidosis have been proposed in animal models, including impaired secretion of both H+ and K+, and downregulation of ammonia transporters in the collecting ducts (Kaneko et al., 2022). In humans, activation of the sodium- chloride cotransporter in the distal tubular cells leads to an impaired negative potential in the collecting duct lumen, and manifests clinically as hyperkalemic RTA and hypertension. Specific differences exist between the two most frequently used CNIs. Cyclosporin causes renal tubular acidosis by blocking the peptidyl prolyl cis-trans isomerase activity through a cyclophilin- dependent mechanism. Such inhibition may cause distal renal tubular acidosis (Watanabe et al., 2005). Tacrolimus, on the other hand, has been noted to affect major transport proteins that are involved in acid base homeostasis in the proximal and distal tubules, including endothelin 1 and H + - ATPase transport protein (Mohebbi et al., 2009). This dose dependent tubular toxicity may be reversed with reduction in the medication dose (Mohebbi et al., 2009).

Rejection can cause tubulitis and ischemic tubular dysfunction, affecting H + ATPase activity and anion exchanger leading to RTA (Mookerjee et al., 1969; Batlle et al., 1981). However, the data proving the association of rejection with metabolic acidosis is somewhat conflicting (Schwarz et al., 2006; Keven et al., 2007). There is reason to speculate that a feedback loop generated from local acidic environments can modulate immune responses, resulting in increased phagocytic activity of macrophages, thus enhancing inflammation (Riemann et al., 2016). Other medications used in the post-transplant period, including the antibiotic Trimethoprim—Sulfamethoxazole, can cause distal type 4 RTA (Pochineni and Rondon-Berrios, 2018).

Lastly, hyperkalemia, a common occurrence post kidney transplantation (generally related to medications, delayed graft function, potassium intake, etc) also contributes to development of acidosis. Hyperkalemia decreases ammonia generation and transport in proximal tubule and collecting duct respectively, leading to impaired ammonia excretion and subsequent metabolic acidosis (Harris et al., 2018). Of the post-transplant immunosuppressants, CNIs are particularly implicated in the development of hyperkalemia by suppression of mineralocorticoid receptor transcriptional activity, leading to signs of hypoaldosteronism (Deppe et al., 2002).

## Complications of metabolic acidosis in kidney transplant recipients

### Association with graft failure

Chronic metabolic acidosis that persists after kidney transplantation may be detrimental to allograft survival. In a large cohort of adult kidney transplant recipients followed for about 62 months (14,271.3 person-years in total), metabolic acidosis defined as the serum total CO_2_ level less than 22 mmol/L at three months post-transplant was associated with increased risk of graft loss and death censored graft failure (Park et al., 2017). Similar results were reported in other studies (Wiegand et al., 2019); however, others have failed to reproduce these findings (Schulte et al., 2019). This points towards the need for rigorously designed trials, focused on metabolic complications following kidney transplantation, as an adjunct to immunological therapies, with the ultimate goal to enhance graft and overall survival.

### Anemia

Post transplantation anemia is common in kidney transplant patients with prevalence being reported around 30–50% of the patients (Yabu and Winkelmayer, 2011; Gafter-Gvili and Gafter, 2019). The pathogenesis appears to be multifactorial, including iron deficiency, reduced graft function, immunosuppressive medications, and erythropoietin resistance secondary to inflammatory state (Yabu and Winkelmayer, 2011). The correlation between metabolic acidosis and anemia has been shown in both hemodialysis and transplant patients (Yorgin et al., 2002). The exact mechanism remains unclear; however, it has been hypothesized that a rightward shift in the oxygen—hemoglobin dissociation curve resulting in downregulation of erythropoietin receptors could be one of the culprits (Ambuhl, 2007).

### Bone disease

Metabolic bone disease after kidney transplantation is due to a host of pathophysiological processes and can take the form of either high or low bone turnover. Acidosis can reduce synthesis of 1,25 (OH)_2_ vitamin D_3_ by proximal tubule, increasing calcium excretion and serum PTH levels, thus promoting bone resorption (Jaeger et al., 1987; Alpern and Sakhaee, 1997; Yakupoglu et al., 2007; Zhang and Chouhan, 2012). *In vitro* acidic environment has been shown to increase resorptive activity of osteoclasts (Murrills et al., 1993). Additionally, pre-transplant bone disease may be worsened by the development of post-transplant acidosis and concomitant use of immunosuppressive regimen, especially corticosteroids and CNIs. As a consequence, close to 9% of bone mineral density may be lost at 18 months post-transplant (Julian et al., 1991), therefore increasing the risk of bone fractures in this patient population (Chiu et al., 1998).

### Frailty and sarcopenia

Individuals with advanced kidney disease often have reduced muscle mass and exercise tolerance (Painter et al., 1986; Dobre et al., 2015; Dubey et al., 2020). Physical capacity and exercise tolerance increases only slightly after kidney transplantation, and this remains an active area of research. Metabolic acidosis has been shown to be one of the contributing factors to sarcopenia after kidney transplantation. Acidosis induces muscle catabolism and inhibits muscle protein synthesis (May et al., 1987; Bailey et al., 2006). It has also been associated with higher serum cortisol levels; the rate of protein catabolism is indirectly associated with serum bicarbonate levels and directly to serum cortisol levels (Garibotto et al., 1994). Correction of acidosis with serum bicarbonate supplementation in chronic kidney disease has shown to decrease proteolysis and improve muscle mass (Reaich et al., 1993; Dubey et al., 2020), but these outcomes have not been studied in kidney transplant recipients. In addition, post kidney transplant patients have been known to have hypophosphatemia. This is largely due to inappropriate renal excretion of phosphorus related to multiple factors including hyperparathyroidism, metabolic acidosis, and tubular dysfunction (Guntupalli et al., 1982; Ghanekar et al., 2006). Increased phosphaturia can result in depletion of intramuscular ATP stores, resulting in sarcopenia.

### Mortality

Metabolic acidosis has been shown to be an independent risk factor for all-cause mortality in CKD (Navaneethan et al., 2011; Raphael et al., 2011; Raphael et al., 2016). These findings were replicated in kidney transplant population. In a single center observational study, comparing kidney transplant recipients with serum bicarbonate 24-25.9 mEq/L, those with serum bicarbonate levels less than 24 mEq/L at one year post transplantation had increased risk of cardiovascular events and all-cause mortality (Djamali et al., 2019). The direct role of metabolic acidosis on increased mortality remains unclear, however worsening cardiovascular disease, including altering the heart contractile function and lowering the trigger for arrhythmias in the setting of acidosis may explain this observed association.

## Metabolic acidosis treatment in kidney transplant recipients

There is a paucity of evidence attesting to the benefits of metabolic acidosis correction in kidney transplant recipients. In fact, in a large retrospective study of 4741 kidney transplant recipients sodium bicarbonate therapy was associated with higher risk of graft failure (Schulte et al., 2019). However there were significant limitations of this study, including the reliance of health insurance data to define the study groups, and the lack of laboratory values to assess allograft function and acid base status.

In a small randomized controlled trial aimed to investigate the effect of sodium bicarbonate therapy on vascular endothelial function in 20 kidney transplant recipients (Bohling et al., 2021), the sodium bicarbonate therapy was considered safe and not associated with increased blood pressures, weight gain, or change in inflammatory markers. A larger trial, looking at similar outcomes in kidney transplant recipients is underway (NCT05005793). The results of Preserve—Transplant study, a multi-center randomized controlled trial designed to investigate the effect of sodium bicarbonate therapy in preserving kidney allograft function and slowing progression of chronic kidney disease in kidney transplant recipients over a period of two years (Wiegand et al., 2018) are eagerly awaited (NCT03102996).

Data on dietary fruits and vegetable consumption as a measure of metabolic acidosis correction in kidney transplant recipients is even scarcer. In CKD, a diet rich in fruits and vegetables favorably affects acid-base metabolism, neutralizes diet-induced acid, and has been shown to be cardio-protective (Goraya et al., 2021). In a prospective cohort of 400 kidney transplant recipients, consumption of a vegetable enriched diet was associated with lower cardiovascular and all-cause mortality (Sotomayor et al., 2020). We propose that the reduced dietary acid load directly derived from a high fruits and vegetables diet contributes to the reduced cardiovascular risk, however the acid base parameters pre- and post-therapy were not reported (Sotomayor et al., 2020).

## Conclusion

Metabolic acidosis is an understudied, highly prevalent complication in kidney transplant recipients. In addition to mechanisms described in metabolic acidosis of CKD, calcineurin inhibitors play a central role in its development and maintenance, through dysregulation of tubular transport proteins involved in acid load handling. Additional research aimed to unfold the mechanisms and consequences of metabolic acidosis present post kidney transplantation will help inform future large intervention trials designed to correct it, in an attempt to prevent allograft loss and improve overall survival in kidney transplant recipients.
